# Factors impacting perceived safety among staff working on mental health wards

**DOI:** 10.1192/bjpo.bp.117.005280

**Published:** 2017-09-05

**Authors:** Alina Haines, Andrew Brown, Rhiannah McCabe, Michelle Rogerson, Richard Whittington

**Affiliations:** **Alina Haines**, PhD, Department of Health Services Research, University of Liverpool, Liverpool, UK; **Andrew Brown**, MSc, Forensic Personality and Autism Spectrum Disorder Assessment and Liaison Team, Mersey Care NHS Foundation Trust, Rainhill, UK; **Rhiannah McCabe**, MSc, Department of Health Services Research, University of Liverpool, Liverpool, UK; **Michelle Rogerson**, PhD, Applied Criminology Centre, University of Huddersfield, Huddersfield, UK; **Richard Whittington**, PhD, Department of Health Services Research, University of Liverpool, Liverpool, UK

## Abstract

**Background:**

Safety at work is a core issue for mental health staff working on in-patient units. At present, there is a limited theoretical base regarding which factors may affect staff perceptions of safety.

**Aims:**

This study attempted to identify which factors affect perceived staff safety working on in-patient mental health wards.

**Method:**

A cross-sectional design was employed across 101 forensic and non-forensic mental health wards, over seven National Health Service trusts nationally. Measures included an online staff survey, Ward Features Checklist and recorded incident data. Data were analysed using categorical principal components analysis and ordinal regression.

**Results:**

Perceptions of staff safety were increased by ward brightness, higher number of patient beds, lower staff to patient ratios, less dayroom space and more urban views.

**Conclusions:**

The findings from this study do not represent common-sense assumptions. Results are discussed in the context of the literature and may have implications for current initiatives aimed at managing in-patient violence and aggression.

**Declaration of interest:**

None.

**Copyright and usage:**

© The Royal College of Psychiatrists 2017. This is an open access article distributed under the terms of the Creative Commons Attribution (CC BY) license.

Safety at work is a fundamental requirement for employers in healthcare and beyond. A subjective sense of safety while working in mental health settings enables the maximum scope for building therapeutic relationships and working effectively with patients. It is important to know what factors from the physical and relational environment on mental health wards are important in generating and maintaining a sense of safety for staff. Such factors may vary between forensic wards and those providing acute care to those without criminal offences, given the different dynamics in the two settings. Forensic settings by definition tend to be populated by a high proportion of people with a history of violent offences but non-forensic wards can feel unsafe because of the acuity or mental illness in the early stages of admission and the high rates of admission and discharge.

Ward aggression and perceived safety are multifaceted phenomena, resulting from a complex interaction between individual (i.e. staff and patient characteristics) and contextual features (i.e. ward/physical environment).^1–4^ In addition to the individual characteristics of patients who have been evidenced to be linked to violence in mental health settings,^5^ there is a growing interest in the role that the physical environment plays in moderating or facilitating violent outcomes in healthcare settings. The physical environment may have a direct influence on safety outcomes in that it is unsafe in various ways (e.g. poor sight lines). But an employee’s sense of safety is also likely to reflect various organisational factors related to safety management and climate which interacts with the overall physical environment. Safety climate^6^ and violence climate^7^ are aspects which have been studied in other work settings and could contribute to safety perceptions on mental health wards. Workplace safety climate includes the perceived commitment to safety and injury avoidance among staff in an organisation. Violence climate is a specific form of safety climate that focuses on perceptions of management attention, concern and policies designed to keep staff safe from violence that has been found to be directly related to safety outcomes.^7^ These organisational factors – or the wider concept of ward culture – have been shown to have a key role in the decision-making regarding the management of in-patient aggression.^8–10^

## Objectives

Surprisingly, in contrast with the growing policy investment in ‘evidence-based’ design of healthcare facilities, research regarding the impact of design on treatment outcomes is inconclusive.^11^ More evidence is needed to improve our understanding of the factors that increase the sense of safety among staff working in these environments which may help us identify and implement appropriate strategies to help manage aggression on the wards. This study aims therefore to ascertain which features across the physical environment, organisational climate and violence are associated with perceived safety among staff. We acknowledge that, given the methodological issues, variables identified as associated may not necessarily be direct predictors of perceived staff safety (unless empirically proven) but may provide an indication of salient factors.

## Method

### Research question

What are the predictors of perceived safety among staff working on mental health wards?

### Design and setting

To answer this question, we used a cross-sectional design surveying 101 forensic and general adult mental health wards across 16 hospitals/units and 7 English National Health Service (NHS) trusts between May 2014 and May 2015. Wards included forensic, general adult, learning disability and psychiatric intensive care units (PICUs). Child and adolescent mental health services (CAMHS) were not chosen to participate because of their limited number and practical issues which would have added subsequent delays to the study.

These trusts were purposively selected through established contacts to cover the NW, NE and SW of England (*n*=4, *n*=1 and *n*=1, respectively) and London (*n*=1) to capture differences across regions.

**Fig. 1:**
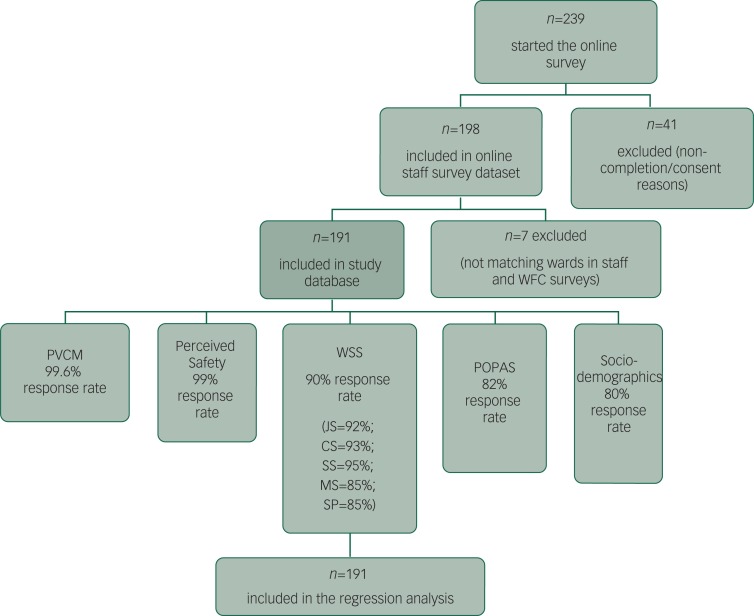
Flowchart of study participants included in the analysis.

POPAS, Perceptions of Prevalence of Aggression Scale; PVCM, Perceived Violence Climate Measure; WFC, Ward Features Checklist; WSS, Work Safety Scale.

An online staff survey (Smart Survey: https://www.smartsurvey.co.uk/) was used to explore staff’s perceived experiences of aggression over the last year, feelings of safety at work and understanding of their organisation’s safety culture. Staff on all wards visited by the research team were invited to take part. The survey took approximately 20 min to complete and was comprised of the perceived safety and violence climate measures described below. Response formats were predominantly Likert scales with minimum free text responses. It is estimated that approximately 1867 staff received the invitation to take part. The response rate was relatively low, with an average of three respondents per ward.^1–16^ In total, 191 respondents were included in the analysis (48 were excluded on the basis of non-completion and lack of appropriate consent) (see Fig. 1 for flowchart of included participants).

National Research Ethics Service permission was not required as participants were all members of staff. Research governance approval was obtained for all participating trusts and researchers were provided with letters of access prior to their visit. The University of Liverpool research ethics committee also approved and governed the research study (ref: IPHS-1314-268).

### Independent variables

#### Physical environment characteristics of the mental health ward: the WFC

The Ward Features Checklist (WFC) was developed by the research team consisting of features identified within the current academic literature and in consultation with clinicians and specialists with experience in the design of mental health wards (available on request from the authors). Components of the newly constructed WFC were refined following a pilot exercise on one ward to determine its feasibility and validity. Inter-rater agreement was inadequate, and a decision was subsequently made for two researchers to collectively gather data from each ward.

The WFC comprises 49 variables covering:

- 2 research/data collection-related variables (i.e. researchers; date and time of extraction)
- 7 general characteristics for the ward/unit (i.e. name of site and ward, function of the ward and gender, average length of stay, number of patients present, number of patients in the building and number of full-time staff per shift) and
- 40 physical environment characteristics (architectural, ambient and interior features).

### Perceived safety and violence climate: the WSS, POPAS and PVCM scales

#### Work Safety Scale

The Work Safety Scale (WSS) is a 50-item validated scale that has been shown to reliably measure five distinct constructs with regard to staff’s perceptions of work safety: (1) job safety, (2) co-worker safety, (3) supervisor safety, (4) management safety practices and (5) satisfaction with safety policies/programmes. Each of these scales has been shown to have a high degree of internal consistency (above 0.87), and there is evidence of convergent and discriminant validity for the WSS.^12^ Respondents are asked to rate their agreement on a 5-point Likert scale, from ‘strongly disagree’ to ‘strongly agree’. Higher scores indicate a stronger sense of perceived safety at work on each of these dimensions.

#### Perceptions of Prevalence of Aggression Scale

A modified version of the Perceptions of Prevalence of Aggression Scale (POPAS) was used to assess staff’s experiences of violence and aggression over the last 12 months. The POPAS has a Cronbach’s alpha of 0.86, indicating good internal consistency.^13^ This scale has been modified to include only the questions regarding staff’s experience of aggression, in its different forms. Staff were asked to rate their experiences on a 5-point scale, ranging from ‘never’ to ‘frequently’, as well as to estimate the number of each incident type in the preceding year. There were a number of problems with the numerical estimates provided by respondents; in particular, estimates were missing for around 40 to 50% of respondents on each of the 10 incident types, and other responses were vague and not possible to quantify (e.g. almost every shift, countless and on occasion). For these reasons, a revised POPAS score was devised from the 10 scaled questionnaire items. The modified POPAS has a Cronbach’s alpha of 0.925 indicating that the revisions have not been detrimental to internal consistently.

#### The Perceived Violence Climate Measure

The Perceived Violence Climate Measure (PVCM) was used to assess violence climate as perceived by the staff working on the mental health wards. The scale comprises seven questions on management attitude, concern and employee safety policies. Responses are in a ‘yes/no/don’t know’ format. The instrument is reported to have satisfactory internal consistency.^7^

#### Socio-demographics

Respondents were also asked to provide information regarding their gender, age and role within the health service.

### Actual safety: recorded incidence of patient aggression on the wards

This included the number of incidents involving verbal and physical aggression, as well as property damage on the ward and number of patients responsible for the incidents. Official electronic trust records were used to collect data on patient aggression. The trusts provided anonymised aggregate data (per ward) regarding aggressive incidents on the respective wards in the 6 months prior to the completion of the WFC.

### Outcome measure/dependent variable

The outcome measure was staff perceptions of safety at work (PSW) measured by a questionnaire item: ‘Please indicate how safe do you feel while at work on the ward on a scale of 1 to 10, “1” being not at all safe and “10” being very safe’.

### Data analysis

#### Categorical principal component analysis

As noted above, the WFC captured 47 variables relating to the ward environment. In order to reduce the complexity of the regression model, it was desirable to reduce the number of dimensions that indicated ward characteristics. This was achieved by conducting a categorical principal component analysis (CATPCA).^14^ This method was used to identify WFC variables that were highly correlated, that is, features that have a strong tendency to occur together. These variables were then grouped and converted into component dimensions. These dimensions replaced the individual variables in the regression model. The advantage of CATPCA over traditional PCA is that it does not assume linear relationships between variables and allows the inclusion of variables with different levels of measurement. CATPCA has been conducted on similar data relating to ward characteristics.^15^

The CATPCA was conducted on the sample of 101 wards in which the WFC was completed. Variables were excluded from the CATPCA (and the subsequent regression model) if there was low variation across the wards of the sample (i.e. where 85% or more wards returned the same value). The analysis identified two dimensions with eigenvalues greater than 1. The first of these dimensions was constructed from six WFC variables, and the second from seven WFC variables. Together, the two dimensions explained 44% of the variance in the WFC. Two subscale scores were created, one for each dimension, which were then related to the outcome variable in a regression model. The remaining WFC variables that were not loaded on either of the two dimensions were entered into the regression model as individual variables.

The first dimension (eigenvalue=3.353) related to the *staffing and space on the wards*. Six variables were loaded on to the component so that a ward scoring highly on this dimension can be interpreted as having (1) fewer beds, (2) higher staff–patient ratios during the dayshift, (3) higher staff–patient ratios during the nightshift, (4) more day room space per patient, (5) more bedroom space per patient and (6) more toilets per patient.

The second dimension (eigenvalue=2.314) related to *comfort and facilities on the ward*. Seven variables were loaded on to this component such that a high score on this variable indicates (1) higher indoor temperature, (2) quieter noise levels, (3) fewer rooms open to patients during the day, (4) the opportunity for patients to participate in games with other patients – measured as a binary, yes/no variable, (5) occupational therapy, (6) the type of flooring and (7) below full-capacity operation.

### Regression model

An ordinal regression model was fitted to analyse the relationship between the independent variables and PSW. The analysis was conducted on a data-set that contained responses from 191 staff members across 60 different wards. Non-response to the staff questionnaire meant that the remaining 41 wards were excluded from this analysis.

The hierarchical nature of the data-set, with staff members clustered within wards, suggests that a multilevel model^16^ would have been advantageous. However, the number of wards where only one staff member returned a questionnaire was high (*n*=22), precluding a robust multilevel analysis.

The independent variables tested were (1) staff level variables including gender, age and role, (2) staff perceptions of workplace safety and violence climate as measured by WSS, POPAS and PVCM, (3) general ward characteristics (forensic/non-forensic and ward function), (4) the WFC including the two dimensions created by the CATPCA and the remaining individual checklist variables and (5) the numbers of reported incidents on each ward.

Independent variables were entered into the model by forward selection, adding a variable one at a time and examining its contribution to the prediction of the dependent variable. Variables that made a significant contribution (*P*<0.05) remained in the model and insignificant variables were removed.

## Results

### Descriptive statistics

#### Ward characteristics

The ward sample consisted of 60 psychiatric wards (34 forensic and 26 non-forensic) with at least one respondent to the safety questionnaire pack.

Most (92.3%) of the non-forensic wards provided acute services to working age or older adults, and the majority of the forensic wards (58.8%) were medium secure with some high secure and low secure services as well. All forensic wards were single sex (80% male) but the non-forensic wards were equally split between female only, male only and mixed. On average, there were 5.5 (s.d.=1.73) staff working per day shift per ward, whereas the average number of patients per ward was 11.81 (s.d.=4.74) for the forensic settings and 14.85 (s.d.=5.95) for the non-forensic setting. The average staff–patient ratio during day shifts was significantly higher in forensic wards (mean=0.48, s.d.=0.26) than in non-forensic wards (mean=0.38. s.d.=0.23) (Mann–Whitney *U*-test 394.0, *P*=0.007). Night shift averages were 0.34 (s.d.=0.21) and 0.31 (s.d.=0.20) for forensic and non-forensic wards respectively, this difference was not statistically significant. The average number of beds was significantly lower in forensic wards (mean=13.44, s.d.=3.97) compared to non-forensic wards (mean=15.62, s.d. 5.42) (Mann–Whitney *U*-test 911.5, *P*=0.026).

Rates of reported violence in the past 6 months per bed were higher on the non-forensic wards. The difference in rates for physical aggression (forensic wards 1.4 (s.d.=2.6), non-forensic wards 4.0 (s.d.=5.6)) was statistically significant (Mann–Whitney *U*-test 926.5, *P*=0.02). Rates of verbal aggression and property damage were also higher on the non-forensic wards (these differences were not statistically significant).

Eleven wards (27.5% of the 40 for which data were available) had been constructed prior to 1990 but over two-thirds of wards in both forensic and non-forensic settings had been through refurbishment in the preceding 5 years and over 90% of wards in both settings had been redecorated in the preceding 5 years. The physical environment characteristics of wards are presented in Table 1. It can be seen that forensic wards were larger with higher ceilings and more diverse colour schemes. Forensic patients had better toilet facilities and greater control over locking their room doors but less control over the ward temperature. Non-forensic wards had greater daylight and more windows.

**Table 1:** Descriptive statistics derived from the Ward Features Checklist

	Type of ward	
	Forensic (*n*=34)	Non-forensic (*n*=26)
Ward Features Checklist: continuous variables (mean, s.d.)		
Total physical space (m^2^)	283.3 (153.4)	208.5 (99.8)
Common useable indoor space (dayroom, m^2^)	109.2 (78.6)	63.4 (26.6)
Common useable outdoor space (accessible through ward, m^2^)	116.5 (63.4)	100.2 (69.7)
Maximum ceiling height	4.6 (3.3)	3.1 (1.1)
Minimum ceiling height^a^	3.8 (2.3)	2.6 (0.2)
Daylight level inside (Lux)	95.3 (81.9)	123.6 (211.8)
Average temperature inside (C) (across 3 time points)	22.5 (2.4)	22.6 (2.1)
Average noise level inside (dB)^a^ (across 3 time points)	67.0 (7.0)	66.8 (9.9)
Categorical variables (*n*,%)		
Patient en-suite toilet available	27 (79.4)	15 (57.7)
Staff toilet available	27 (79.4)	23 (88.5)
Laminate/timber flooring	28 (82.4)	23 (88.5)
Yellow colour scheme (mainly)	21 (61.8)	21 (80.8)
Number of windows		
Two windows	10 (29.4)	5 (20.8)
Three windows	6 (17.6)	5 (20.8)
Three plus	18 (53.0)	14 (58.4)
View (from window)		
Greenery	6 (19.4)	8 (33.3)
Concrete/building	5 (16.1)	5 (20.8)
Mixed	20 (64.5)	11 (45.8)
Entertainment available		
TV/DVD	32 (94.1)	23 (88.5)
Computer games	29 (85.3)	22 (84.6)
Occupational therapy activities/recreational	16 (47.1)	8 (30.8)
Social games	23 (67.6)	14 (53.8)
Floor level location		
Ground	30 (88.2)	20 (76.9)
First floor	4 (11.8)	5 (19.2)
Second floor	0 (0)	1 (3.8)
Patient can open window	22 (64.7)	20 (76.9)
Patient can control temperature^b^	2 (5.9)	17 (65.4)
Patient can lock bedroom	31 (91.2)	17 (65.4)

a Statistically significant difference forensic versus non-forensic wards: Mann–Whitney *U*-test=300.0, *P*=0.000.

b Statistically significant difference forensic versus non-forensic wards: Chi square=4.734, *P*=0.030.

#### Staff characteristics

There were 191 staff respondents from these 60 wards. Ninety-five were women (49.7% of those for whom gender data were available) and 51 were men (26.7%). The majority were either qualified nurses (45.5%) or nursing assistants (30.4%). Of the 153 staff who provided their age, the most frequent age category was 25–34 (27.2%).

### Staff perceptions of safety

The safety perception outcome variable was banded into quartiles (5 or less; 6–7; 8; and 9–10) with similar numbers of respondents. This reduced the statistical problems created by the low number of responses for some of the original values (particularly those at the lower end of the scale). A higher proportion of forensic staff reported feeling relatively unsafe at work (26.3% forensic staff and 20.5% non-forensic staff) and conversely a much higher proportion of non-forensic staff reported feeling safe at work (30.1% of non-forensic staff and 17.8% of forensic staff). This difference was not statistically significant (Mann–Whitney *U*-test 4691.5, *P*=0.24). Table 2 reports the scores for each subscale on the individual staff-level predictor variables

**Table 2:** Descriptive statistics derived from work safety climate instruments

	Staff on forensic wards	Staff on non-forensic wards
Perception of safety at work (1 to 10) (%)	*n*=118	*n*=75
5 or less	26.3	20.5
6–7	21.2	27.4
8	34.7	21.9
9–10	17.8	30.1
Work Safety Scale (WSS) (mean, s.d.)	*n*=118	*n*=75
Job safety	2.9 (0.7)	3.1 (0.8)
Co-worker safety^a^	3.9 (0.6)	4.1 (0.6)
Supervisor safety	3.8 (0.6)	3.8 (0.7)
Management safety practices	3.6 (0.6)	3.7 (0.8)
Safety policies and programmes	3.7 (0.6)	3.8 (0.7)
WSS total score^b^	3.6 (0.5)	3.7 (0.5)
Perceptions of Violence Climate Scale (PVCM) (% ‘Yes’)	*n*=118	*n*=75
Does your employer provide assault/violence prevention training?	99.2	98.3
Does your employer provide assault/violence prevention policies and procedures?	98.3	98.6
Are there procedures in place in your facility for reporting violence?	100.0	97.3
Does management encourage staff to report physical violence?	99.2	89.0
Does management encourage staff to report verbal violence?^c^	90.7	72.6
Are reports of workplace violence from other employees taken seriously by the management?	78.6	75.3
When patients/residents assault staff, does management consider it just a ‘part of the job’?^d^	29.7	30.1
PVCM total score (mode (%))	6 (73.7%)	6 (63%)
Perceptions of the Prevalence of Aggression Scale (POPAS) (% endorsing modal category)	*n*=118 Mode (%)	*n*=73 Mode (%)
Verbal aggression	Frequently (40.0)	Frequently (46.6)
Threatening verbal aggression	Sometimes (26.0)	Occasionally (28.6)
Humiliating aggressive behaviour	Occasionally (25.7)	Occasionally (28.1)
Provocative aggressive behaviour	Sometimes (28.7)	Sometimes (29.3)
Passive aggressive behaviour	Occasionally (28.0)	Sometimes (37.5)
Aggressive splitting behaviour	Frequently (29)	Sometimes (43.9)
Threatening physical aggression	Sometimes^e^ (28)	Sometimes^f^ (31.6)
Destructive aggressive behaviour	Occasionally (34)	Occasionally (40.4)
Physical violence without physical injury	Occasionally (27.3)	Occasionally^g^ (33.3)
Physical violence leading to injury	Never (76.8)	Never (78.9)
POPAS total score (mean, s.d. (available range 1–5))	2.8 (0.97)	2.8 (0.88)

a Significant difference forensic versus non-forensic wards: Mann–Whitney *U*-test=4573.5, *P*=0.024.

b Significant difference forensic versus non-forensic wards: Mann–Whitney *U*-test=4592.0, *P*=0.037.

c Significant difference forensic versus non-forensic wards: Chi square=0.82, *P*<0.05.

d Reverse scored.

e Multiple mode: equal frequency for ‘occasionally’.

f Multiple mode: equal frequency for ‘occasionally’.

g Multiple mode: equal frequency for ‘never’.

There was little variation across the dimensions of the WSS with the majority of staff members answering ‘agree’ to positively worded safety statements and ‘disagree’ to negatively worded statements. Exceptions to this were the job safety dimension with respondents tending to agree that their job was ‘dangerous’, ‘hazardous’, ‘risky’ and ‘scary’ and that they could ‘get hurt easily’. WSS scores are generally higher for staff working on non-forensic wards compared with forensic settings, but this difference was only significant for the co-worker safety dimension (Mann–Whitney *U*-test 4573.5, *P*=0.024) and the overall WSS score (Mann–Whitney *U*-test 4592.0, *P*=0.037).

### Staff experience of aggression

On four of the seven PCVM items, there was minimal variation across the sample, with 90% or more respondents answering ‘yes’. This indicates positive perceptions of the provision of violence training, prevention policies and procedures, facilities for reporting violence and encouragement to report physical violence. Around 80% of respondents stated that their management encouraged staff to report physical violence; however, there was a significant difference of 10 percentage points between staff working on forensic wards and those working on non-forensic wards on this item (chi square 10.58, *P*=0.004). Although still a large majority (77% of all staff), a smaller proportion of respondents stated that workplace violence from other employees was taken seriously. Finally, only 30% of respondents believed that management considered that assaults on staff by patients was just ‘part of the job’. The difference between forensic and non-forensic wards on the total PVCM score was not statistically significant.

The results from the POPAS in Table 2 reveal that the incident types the staff members were confronted by most frequently were verbal aggression and aggressive splitting behaviour. The majority of respondents stated that they never experienced physical violence leading to injury. There were some marginal differences between staff working on forensic and non-forensic wards but these were not statistically significant. A higher proportion of staff on forensic wards experienced verbal aggression and aggressive splitting behaviour frequently compared with those on non-forensic wards.

### Factors impacting perceived safety among staff working on psychiatric wards

Table 3 summarises the results of the model to predict the outcome measure staff PSW. The table presents the results as proportional odds ratios (ORs) with 95% confidence intervals and significance values. Proportional odds ratios can be interpreted as the change in the odds of a respondent selecting a higher value on the PSW scale given a unit increase in the independent variable. An OR>1 indicates that as the independent variable increases the odds of a staff member indicating higher PSW also increase. An OR <1 indicates a statistically negative relationship whereby an increase in the independent variable is associated with a decrease in PSW. Features that increased PSW included the total WSS score (OR=5.28), the total PVCM score (OR=1.85) and brightness inside the ward with lights on (OR=1.53). Features that decreased PSW included the ‘staffing and space’ dimension of the WFC generated via CATCPA (OR=0.65), the number of recorded verbal incidents (OR=0.98) and the number of recorded property incidents (OR=0.90). Staff working on wards with views of man-made structures/concrete (OR=0.33) and greenery (OR=0.25) reported lower PSW than staff working on wards with ‘mixed’ views. The ‘comfort and facilities’ dimension of the WFC was not significant.

**Table 3:** Results of ordinal regression for the outcome variable perceived safety at work (*n*=191)

Measures	Proportional odds ratio (95% CI)	*P*
Staffing and space	0.65 (0.48–0.89)	0.007
WSS score	5.28 (2.65–10.51)	<0.001
PVCM score	1.85 (1.14–3.01)	0.013
Recorded number of verbal incidences	0.98 (0.97–1)	0.014
Recorded number of property incidents	0.90 (0.83–0.98)	0.019
Brightness inside the ward with lights on^a^	1.53 (1.09–2.15)	0.014
View from the ward (Base=Concrete/Built up)		
View from the ward=Mixed	0.33 (0.12–0.95)	0.04
View from the ward=Greenery	0.25 (0.08–0.77)	0.016

a Standardised. Link function: Logit. The pseudo *R*^2^ value of Cox and Snell = 0.409. Test of parallel lines *P*>0.05; therefore, the proportional odds assumption was not violated.

These findings suggest that perceptions of safety were *higher* on wards where staff had positive feelings of the workplace safety climate. Both the total WSS score and the PVCM were significant and positively related to PSW. Staff who expressed positive views towards the safety culture and climate on their wards reported more positive PSW. Perceptions of safety were higher on wards that had brighter lighting levels but also on wards that have more beds, lower staff–patient ratios, less day room space, less bedroom space and fewer toilets per patient. Compared with staff working on wards which had a view of built-up, man-made structures (concrete), staff working both on wards with gardens or countryside views (greenery) and those with mixed views had more negative PSW. Perceptions of safety were lower on wards with higher reported levels of verbal incidents and property incidents. The POPAS measure of self-reported exposure to incidents were also inspected and, although there were bivariate associations between increased experience of harassment, assaults and threats on the ward, these were no longer significant once included in the multivariate model and other variables were controlled for. Other variables that were not significant predictors of PSW included staff characteristics (gender age, role), type of ward (forensic *v*. non-forensic) and physical ward features such as number of windows and ward colour.

## Discussion

### Main findings

Findings indicate that staff’s perceptions of safety were influenced by some aspects of the wards’ design characteristics and presence of aggression in the workplace. Interestingly, some of the predictors of PSW identified by the regression model appear to run counter to common sense assumptions with ‘green’ views, high staff–patient ratios and spacious wards being among the factors associated with *more negative* perceptions of safety, and higher levels of reported verbal and property incidents predicting *more positive* perceptions of safety.

According to existing literature, mental health nurses have not always felt supported to report aggressive incidents that take place on the ward.^17,18^ A variety of reasons for this have been suggested, for example, because of incident reports being disseminated outside of the nursing domain and scrutinised with a more business-like, bureaucratic approach.^19^ Despite previous findings, this study found the majority of staff were encouraged to report such incidents and that wards with higher rates of reported verbal and property incidents predicted more positive PSW. High incident rates may therefore be a reflection of a positive safety culture in which management encourage their staff to report incidents, rather than simply reflecting a ward that experiences more incidents.

The finding that greater bed numbers and staff–patient ratios contribute to negative PSW contradicts previous research and indeed what common sense would suggest. Staffing levels have previously been found to be a key factor in determining a safe service,^20^ and some studies have reported correlations between high ward occupancy,^21^ crowding^22^ and high levels of aggressive incidents. One explanation for the incongruity here could be that staff working on more crowded wards have increased resilience and thus maintain a sense of safety despite encountering high levels of risk. Research has previously found high levels of resilience in mental health nurses^23^ and specifically resilience around assaults in mental health staff.^24^ It is therefore possible that staff on crowded wards may have a greater tolerance for aggressive incidents or that crowded wards have more experienced staff. This could also be linked to a culture of minimisation of aggression on busier wards, just to reduce paperwork necessary for recording such incidents. These are, however, only hypotheses at this stage and would require further research to substantiate.

In terms of other predictors, the positive association between ward brightness and feelings of staff safety corroborates the importance of ward light in the existing literature.^25^ Previous research has specified that lighting should be natural^26^ for maximum benefit; however, this was not measured in this study. The benefits of lighting in the literature included improved observations (which in turn improved security) as well as helping with patient’s sleep cycles.^26^ In this study, wards with views of greenery were associated with less safe feelings, in comparison with those with urban views. The literature concurs that visual access to outdoor spaces is important^25–27^ for a number of reasons including opportunities for recovery, activities and social interactions. One hypothesis for the counter-intuitive finding here could be that greenery is more likely in rural surroundings. This may cause staff to feel more isolated and subsequently less safe than in a busier, urban surrounding. Again, this is only a hypothesis and would require further research.

The authors consider this research to be novel in that it contributes to a relatively small existing literature base. A proportion of research claiming to examine staff safety tends to use a proxy outcome measure through the number of aggressive incidents. This study has measured and reported both and indeed suggests that one is not a good proxy for the other as they were negatively associated here. Ward variables, such as noise and light in particular, have been under-researched up until now and it is hoped that the findings of this study can now add to the literature base.

The ward environment is a priority for staff safety,^26^ and it is important that this area continues to be researched. What is clear from the, at times counter-intuitive, findings in this study are the challenges related to studying the therapeutic landscape in its entirety. Mental health wards are comprised of complex relational dynamics, from which it is impossible to differentiate the role of the physical environment in determining staff perceptions of safety from other factors.

### Limitations of study

One should take into account the following limitations when interpreting the results presented here. Staff questionnaires were not returned from all wards and a multi-level analysis could not be conducted as there were not sufficient responses per ward. Some variables may not have held significance in the regression model because of the low sample size compared with the number of dimensions being examined. It should also be noted that some of the variables remain ambiguous, for example, available space. Consequently, some caution is recommended when interpreting the results for this and other ambiguous variables. Some physical ward characteristics found to be significant in other studies did not contribute to the model here. This may be the result of difficulties in developing indicators for important factors and measuring them accurately.

### Implications

The results have demonstrated the importance of an organisational culture that fosters a positive safety climate, ensuring there are relevant polices, that training is provided to staff and that incidents of aggression (of all types) are taken seriously.

These findings have direct implications for broadening the scope of staff training. Existing initiatives, such as No Force First within Mersey Care NHS Foundation Trust, aim to equip staff with the skills to manage patient violence and aggression more effectively, and when possible without coercive intervention. This research has identified additional factors that may influence staff feelings of safety in terms of the ward environment that could affect decision-making around intervention. In terms of the findings of this study, the reality of process that affect staff feelings of safety do not necessarily meet the expectation. Disseminating the results with front-line staff should serve to inform practice and provide further opportunities to contextualise the results. Future research adopting a qualitative methodology may provide some clarity regarding these results and explore suggested hypotheses further.
